# American Medical Association

**Published:** 1886-03

**Authors:** 


### SOCIETY NOTES.

### AMERICAN MEDICAL ASSOCIATION.

PHILADELPHIA; 1400 PINE STREET, S. W. cor. Broad.

THE Thirty-seventh Annual Session will be held in St. Louis, Mo., on Tuesday, Wednesday, Thursday and Friday, May 4, 5, 5 and 7, commencing on Tuesday, at 11 a. m.

"The delegates shall receive their appointment from permanently organized State Medical Societies, and such County and District Medical Societies *as are recognized by representatives in their respective State Societies*, and from the Medical Department of the Army and Navy, and the Marine Hospital Service of the United States.

"Each State, County and District Medical Society entitled to representation shall have the privilege of sending to the Association one delegate for every ten of its regular resident members, and one for every additional fraction of more than half that number: *Provided*, however, that the number of delegates for any particular State, territory, county, city or town shall not exceed the ratio of one in ten of the resident physicians who may have signed the Code of Ethics of the Association.''

Secretaries of Medical Societies, as above designated, are earnestly requested to forward, *at once*, lists of their delegates.

Also, that the Permanent Secretary may be enabled to erase from the roll the names of those who have forfeited their membership, the Secretaries *are, by special resolution*, requested to send him, annually, a corrected list of the membership of their respective Societies.

SECTIONS.

"The Chairmen of the several Sections shall prepare and read, in the general sessions of the Association, papers on the advances and discoveries of the past year in the branches of science included in their respective Sections. * * * *."—*By-Laws*, Art. II, Sec. 4.

*Practice of Medicine, Materia Medica and Physiology:* Dr. J. T. Whittaker, Cincinnati, Ohio, Chairman; Dr. B. L. Coleman, Lexington, Ky., Secretary.

*Obstetrics and Diseases of Women and Children:* Dr. S. C. Gordon, Portland, Me., Chairman; Dr. J. F. Y. Paine, Galveston, Texas, Secretary.

*Surgery and Anatomy:* Dr. Nicholas Senn, Milwaukee, Wis., Chairmann; Dr. H. H. Mudd, St. Louis, Mo., Secretary.

*State Medicine:* Dr. John H. Rauch, Springfield, Ill., Chairman; Dr. F. E. Daniel, Austin, Texas, Secretary.

*Ophthalmology, Otology and Laryngology:* Dr. Eugene Smith, Detroit, Mich., Chairman; Dr. J. F. Fulton, St. Paul, Minn., Secretary.

*Diseases of Children:* Dr. W. D. Haggard, Nashville, Tenn., Chairman; Dr. W. B. Lawrence, Batesville, Ark., Secretary.

*Oral and Dental Surgery:* Dr. John S. Marshall, Chicago, Ill., Chairman; Dr. A. E. Baldwin, Chicago, Ill., Secretary.

A member desiring to read a paper before a Section should forward the paper, or its *title* and *length* (not to exceed twenty minutes in reading), to the Chairman of the Committee of Arrangements, at least one month before the meeting.—*By-Laws.*

*Committee of Arrangements.*—Dr. Le Grand Atwood, St. Louis, Mo., Chairman.

We give the above in full for the information of the Texas Profession, and especially of those who desire to attend the meeting or contribute a paper to any of the Sections.—Ed.

